# The Risk of Cardiovascular Sequelae in Post-Acute COVID-19: Comorbidity or COVID?∗

**DOI:** 10.1016/j.jacadv.2023.100455

**Published:** 2023-07-27

**Authors:** C. Anwar A. Chahal, Bhurint Siripanthong

**Affiliations:** a Center for Inherited Cardiovascular Diseases, WellSpan Health, Lancaster, Pennsylvania, USA; b Barts Heart Centre, St Bartholomew’s Hospital, Barts Health NHS Trust, London, West Smithfield, United Kingdom; c Department of Cardiovascular Medicine, Mayo Clinic, Rochester, Minnesota, USA; d Royal Sussex County Hospital, University Hospitals Sussex NHS Foundation Trust, Brighton, United Kingdom

**Keywords:** heart failure, hypertension, long COVID, post-acute COVID-19

**Figure:**
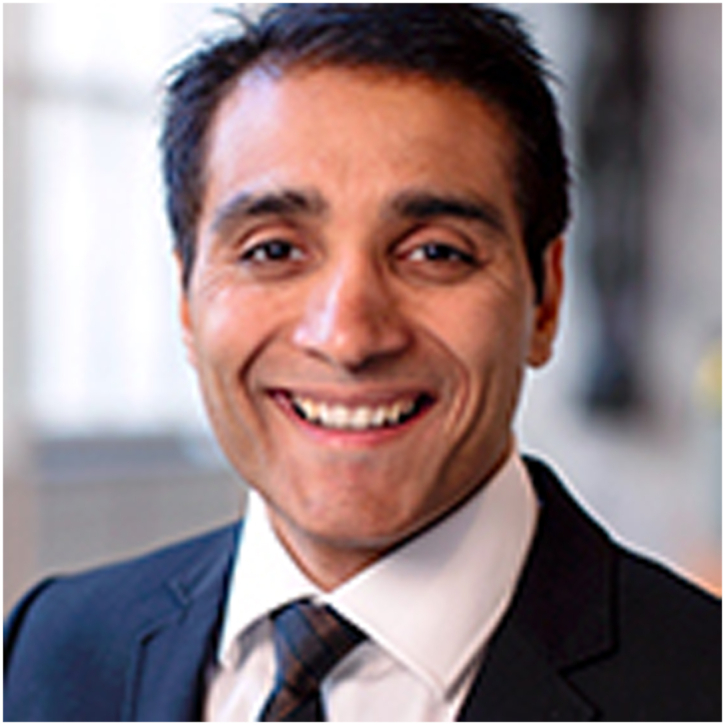

The COVID-19 pandemic, caused by SARS-CoV-2, has had an immense impact on global and individual health. Since the start of the pandemic in late 2019, there have been over 760 million confirmed cases of COVID-19 infection, with nearly 7 million deaths attributable to the disease.^1^ In addition to the acute phase, which refers to the period within 3 months of infection, long COVID or post-acute sequelae of SARS-CoV-2 has been reported in individuals of all ages, backgrounds, and severity of initial infection.^2^ The World Health Organization (WHO) defines post-acute COVID-19 as the continuation or development of new symptoms 3 months after the initial SARS-CoV-2 infection, with these symptoms lasting for at least 2 months without any other explanation. Individuals with post-acute COVID-19 may experience various symptoms, including fatigue, headache, chest pain, depression, and anxiety, among others. Undoubtedly, this has a significant impact on both individual health and the health care systems.^3^

Among the many reported long COVID-19 symptoms, the cardiovascular sequelae have received considerable attention. Dyspnea, palpitations, chest pain, and thromboembolism^4^^,^^5^ are recognized symptoms during the post-acute phase (Figure 1), and various pathophysiological mechanisms have been proposed to explain their occurrence or recurrence. One such proposition is that SARS-CoV-2 infects endothelial cells expressing angiotensin-converting enzyme 2 receptors, leading to cardiovascular endotheliitis.^6^ This causes damage to the vasculature, which can precipitate myocardial infarction, thrombosis, and other cardiovascular sequelae.^7^ Of note, cardiovascular autonomic dysfunction, including postural orthostatic tachycardia syndrome, is also posited to occur in the post-acute COVID period.^8^^,^^9^ However, it remains unclear to what extent these symptoms can be attributed to prior COVID-19 infection. Several reports have used different inclusion criteria to study the cardiovascular impact of post-acute COVID-19, deviating from the WHO definition (symptoms persisting for three or more months after initial infection, lasting at least 2 months).^10^, ^11^, ^12^

**Figure 1:**
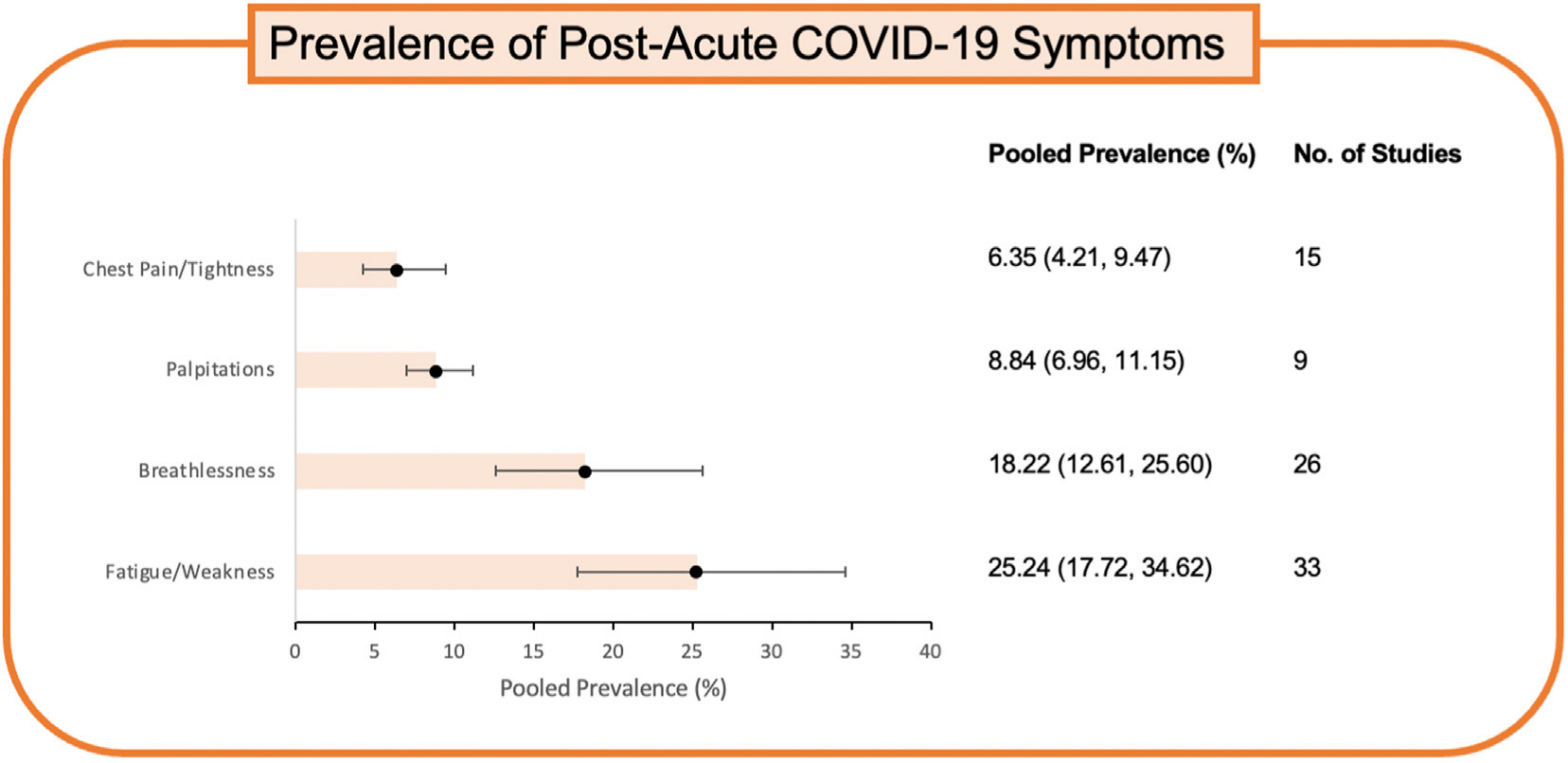
**Cardiovascular Symptoms in Post-COVID-19 Infection Patients** Table shows pooled prevalence in % of the common cardiovascular complaints, namely, fatigue, breathlessness, palpitations, and chest pain in post-acute COVID-19 patients. Modified from O'Mahoney et al.^5^

The current study by McAlister et al^13^ in this issue of *JACC: Advances* evaluates long COVID in the context of hospitalizations or emergency department (ED) visits for cardiovascular complaints (including diabetes mellitus, hypertension, and heart failure) using the strict WHO definition. The authors conducted a retrospective cohort study using data sets from the universal access health care system of Alberta, Canada. They compared all adults in Alberta who had a confirmed COVID-19 infection (by real-time polymerase chain reaction) to matched controls (matched by age, sex, and Charlson comorbidity index score) who tested negative for COVID-19 during the contemporaneous period of March 1, 2020, to June 30, 2021. They assessed the likelihood of ED visits or hospitalization during the post-acute COVID-19 phase (3-9 months postinfection).They found that cases were more likely than controls to have ED visits or hospitalizations for diabetes (1.5% vs 0.7%, *P* < 0.0001), hypertension (0.6% vs 0.4%, *P* < 0.0001), heart failure (0.2% vs 0.1%, *P* = 0.0002), and kidney injury (0.3% vs 0.2%, *P* < 0.0001). The results were even more pronounced when considering only COVID-19 hospitalization survivors compared to the appropriate control group. However, there was no statistical difference in the likelihood of presentation with acute coronary syndrome, stroke, cardiac arrhythmias, or bleeding.

The authors deserve commendation for their comprehensive data collection, which included all cases that tested positive for COVID-19 by real-time polymerase chain reaction (177,892 cases), thereby minimizing selection bias among the Alberta population. Additionally, they addressed a focused question using appropriate statistical tools and adjusted for confounding variables. They also implemented an adequate follow-up period of 6 months (ie, 3-9 months postinfection), fully complying with the WHO definition of post-acute COVID-19 sequelae (ie, long COVID), a feature lacking in many previous publications on post-acute COVID-19 symptoms. Importantly, the article aimed to raise awareness among clinicians regarding the impact of long COVID and the importance of screening COVID survivors for specific cardiovascular complications, such as diabetes mellitus, hypertension, heart failure, and kidney dysfunction. This information could potentially inform public health policymaking, particularly in resource allocation and optimization for the secondary prevention of COVID-19 sequelae.

As a cohort study relying on administrative data, there are several limitations outlined by the authors. For instance, the number of vaccinated individuals was very small (<3%), and the strain of COVID analyzed in this study may differ from the current dominant COVID variants.

In addition to the limitations discussed in the article, another consideration could be to present the episodes of acute presentations as a ratio of pre-existing disease prevalence. For example, despite COVID-19 patients having a higher likelihood of presenting with hypertension in the post-acute phase (0.6% vs 0.4%), the prevalence of pre-existing hypertension is also greater among COVID-19 patients compared to the control group (2.5% vs 1.7%). This trend holds for all statistically significant sequelae studied (heart failure 0.8% vs 0.4%, diabetes mellitus 4.5% vs 2.0%, and renal disease 0.6% vs 0.3%). Therefore, the question arises as to whether the increased likelihood of post-acute presentations can be partially explained as an acute exacerbation of pre-existing conditions. However, the authors demonstrated that when considering only the new diagnosis or incidence of the aforementioned cardiovascular complications, statistically significant results were maintained.

In conclusion, the current study revealed an increase in the number of ED visits and hospitalizations among COVID-19 survivors for cardiovascular sequelae during the post-acute COVID period. A stronger association was observed among COVID-19 hospitalization survivors. However, many aspects of this phenomenon remain unknown, highlighting the need for further research. For example, the pathophysiology or mechanism of long COVID remains unclear, with current evidence showing significant heterogeneity.^14^ Several studies have linked a low or reduced immune response to the initial infection with the occurrence of long COVID. For instance, patients with a low CD4+ and CD8+ response, a high level of autoantibodies, or low SARS-CoV-2 antibody production are more likely to develop long COVID.^15^ Nevertheless, predispositions or risk factors for long COVID are still active areas of research. Lastly, the current screening tools and treatment options for long COVID are inadequate, necessitating further clinical trials to address this expanding public health issue.

## Funding support and author disclosures

The authors have reported that they have no relationships relevant to the contents of this paper to disclose.

## References

[1] WHO (2023).

[2] Carfì A., Bernabei R., Landi F., Gemelli Against COVID-19 Post-Acute Care Study Group (2020). Persistent symptoms in patients after acute COVID-19. JAMA.

[3] Rubin R. (2020). As their numbers grow, COVID-19 “long haulers” stump experts. JAMA.

[4] Nalbandian A., Sehgal K., Gupta A. (2021). Post-acute COVID-19 syndrome. Nat Med.

[5] O'Mahoney L.L., Routen A., Gillies C. (2023). The prevalence and long-term health effects of long Covid among hospitalised and non-hospitalised populations: a systematic review and meta-analysis. EClinicalMedicine.

[6] Varga Z., Flammer A.J., Steiger P. (2020). Endothelial cell infection and endotheliitis in COVID-19. Lancet.

[7] Siripanthong B., Asatryan B., Hanff T.C. (2022). The pathogenesis and long-term consequences of COVID-19 cardiac injury. J Am Coll Cardiol Basic Trans Science.

[8] Dani M., Dirksen A., Taraborrelli P. (2021). Autonomic dysfunction in 'long COVID': rationale, physiology and management strategies. Clin Med.

[9] Fedorowski A., Sutton R. (2023). Autonomic dysfunction and postural orthostatic tachycardia syndrome in post-acute COVID-19 syndrome. Nat Rev Cardiol.

[10] Daugherty S.E., Guo Y., Heath K. (2021). Risk of clinical sequelae after the acute phase of SARS-CoV-2 infection: retrospective cohort study. BMJ.

[11] Cohen K., Ren S., Heath K. (2022). Risk of persistent and new clinical sequelae among adults aged 65 years and older during the post-acute phase of SARS-CoV-2 infection: retrospective cohort study. BMJ.

[12] Al-Aly Z., Xie Y., Bowe B. (2021). High-dimensional characterization of post-acute sequelae of COVID-19. Nature.

[13] McAlister F.A., Nabipoor M., Wang T., Bakal J.A. (2023). Emergency visits or hospitalizations for cardiovascular diagnoses in the post-acute phase of COVID-19. JACC: Adv.

[14] Castanares-Zapatero D., Chalon P., Kohn L. (2022). Pathophysiology and mechanism of long COVID: a comprehensive review. Ann Med.

[15] Davis H.E., McCorkell L., Vogel J.M., Topol E.J. (2023). Long COVID: major findings, mechanisms and recommendations. Nat Rev Microbiol.

